# Evaluating the association of self-reported psychological distress and self-rated health on survival times among women with breast cancer in the U.S.

**DOI:** 10.1371/journal.pone.0260481

**Published:** 2021-12-01

**Authors:** Oluwaseun John Adeyemi, Tasha Leimomi Gill, Rajib Paul, Larissa Brunner Huber

**Affiliations:** 1 Department of Trauma and Orthopaedics, University of Edinburgh, Edinburgh, United Kingdom; 2 Department of Public Health Sciences, University of North Carolina at Charlotte, Charlotte, NC, United States of America; Emory University, School of Public Health, UNITED STATES

## Abstract

**Background:**

Psychological distress and self-rated health status may create additional complexities in patients already diagnosed with breast cancer. This study aims to assess the association of self-report-based assessment of psychological distress and self-rated health on survival times among women with breast cancer diagnoses.

**Methods:**

Seventeen-year data from the Integrated Public Use Microdata Series—National Health Interview Survey (IPUMS-NHIS) were pooled and analyzed. Women who were aged 30 to 64 years old, with breast cancer diagnosis were selected (n = 2,819). The outcome variable was time to death. The independent variables were self-reported assessment of psychological distress and self-rated health. Psychological distress was defined using the Kessler-6 scale while self-rated health was measured on a 3-point Likert scale: Poor, Fair, and Good-to-Excellent (referred to as good for brevity). We computed unadjusted and adjusted hazard ratios (HR) using Cox-Proportional Hazard regression models with sociodemographic characteristics and measures of health care access used as potential confounders. Significance was set at alpha = 0.05.

**Results:**

Women with breast cancer assessed as having psychological distress had 46% (Adjusted HR: 1.46; 95% CI: 1.02–2.09) increased risks of mortality. Also, women who rated their health as poor or fair had a significantly elevated mortality risk (Poor Health: Adjusted HR: 3.05; 95% CI: 2.61–4.69; Fair Health: Adjusted HR: 1.83; 95% CI: 1.43–2.35) as compared to women with good health status.

**Conclusions:**

Self-reported psychological distress and fair and poor self-rated health are associated with reduced survival times among women with breast cancer diagnoses.

## 1. Introduction

In the United States (U.S.), breast cancer is the second most common cancer and the second leading cause of cancer death in women [1–3]. It is estimated that 1 out of every 8 U.S. women has a lifetime risk of developing breast cancer and the incidence of breast cancer in the U.S. has increased by 0.5% per year [2]. Also, about 1 in 39 women die from breast cancer with death rates higher among Black women compared to Whites [2, 3]. Although the death rates have consistently declined among older women, deaths from breast cancer among women less than 50 years have remained steady [2, 4].

Advances in cancer screening, diagnosis, and treatment, have improved the quality of life and five-year survival rates of breast cancer patients in the U.S. [1]. However, these undeniable medical advancements do not eliminate the fears of separation, pain, isolation, and death among breast cancer patients [5]. Psychological distress compounded the direct burden of breast cancer borne by these patients, manifesting commonly with non-specific symptoms of mood disorders, anxiety, and depression [5, 6]. Psychological distress, defined as the overburden or the inability to cope with negative affect-eliciting events [7], is most commonly experienced at the time of breast cancer diagnosis [8, 9], but may persist longer and contribute to the emergence of other chronic co-morbid conditions [9]. A recent study has demonstrated that mortality from other coexisting chronic causes exceeds mortality from primary breast cancer disease [10]. Hospital-based studies have estimated that psychological distress among women with breast cancer diagnosis range widely from 30% to 75% [8, 9].

Indeed, psychological distress may occur before cancer diagnosis, may be due to cancer diagnosis, treatment, or recurrence, or may be unrelated to cancer diagnosis. Irrespective of the temporal relationship of psychological distress and breast cancer, the presence of psychological distress has been associated with increased cancer-related mortality [11]. Several pathways exist between psychological distress and breast cancer-related mortality, one of which is through the effect of cortisol [11, 12]. Increased cortisol leads to increased body adiposity, and decreased physical exercise, which in turn leads to increased peripheral estrogen production [11–15]. Increased peripheral estrogen production increases breast cancer risk and/or its progression or recurrence [13–15]. Psychological distress also increases DNA damage, poor DNA repair, shortened telomeres, and decreased telomerase activity, all of which either promote tumorigenesis or cancer progression [11, 16–19]. Moreover, psychological distress suppresses the immune system by reducing the responses of the natural killer cells and lymphocytes to cancer antigens [11, 20]. These suppressive effects occur through several behavioral and neuroendocrine pathways some of which include poor sleep, increased alcohol consumption, smoking, and increased cortisol, neuropeptides, and catecholamine production via the hypothalamic-pituitary-adrenal axis [11, 21].

In contrast to psychological distress assessment, a provider-based evaluation, self-rated health is a reflection of the psychosocial and self-perceived clinical state [22]. For individuals with breast cancer, perception of health status may be informed by the primary cancer disease state, coexisting breast cancer-related and non-breast cancer-related morbidities, or the combination of all health-related factors. Despite the subjectivity associated with self-rated health, its relationship with mortality, irrespective of cause, is well documented [23–25]. Individuals who rated their health are poor have two-folds increased risks of all-cause mortality compared to those who rated their health as excellent [26]. Among women who died from breast cancer and cancer-related deaths, self-rated health perception declines with increasing life events [27]. These life events may include but are not limited to the presence of coexisting medical conditions, illness and death among family members, a decline in socioeconomic status, marriage and divorce, problems with childbirth or with children, and crime victimization [27].

Irrespective of the metric used in assessing psychological stress and health status, breast cancer diagnosis and treatment remains a stressful event. Earlier studies [28–30] have reported that psychological distress, expressed as non-specific anxiety, depression, and mood disorders, may increase the mortality among women with breast cancer. Additionally, the complications from surgery, systemic therapy, and radiotherapy and the potential of recurrence may induce negative effects of neuroendocrine stress hormones [31, 32]. With improvements in breast cancer diagnosis and treatment, it is expected that the survival rates of women with breast cancer will keep increasing. However, psychological distress may undermine the survival times of women with breast cancer diagnoses. Also, women with breast cancer may observe a decline in health status and their self-rated health may indicate negative health outcomes. It is unknown to what extent psychological distress and self-rated health reduce the survival times of women with breast cancer. Understanding the extent to which psychological distress and self-rated health associates with breast cancer survival may inform the need for screening and psychosocial interventions among women with breast cancer diagnoses. This study aims to assess the association of self-reported psychological distress and self-rated health on the survival times among women with breast cancer.

## 2. Methods

### 2.1. Study population

We pooled 17 years of data, from 1998 to 2014, from the Integrated Public Use Microdata Series (IPUMS) of the National Health Interview Survey (NHIS). IPUMS is a publicly available platform that aggregates data from the NHIS, accounting for changes in survey questions across time, and presenting NHIS variables consistent across time in downloadable formats [33]. The NHIS is one of the oldest and largest surveys in the U.S. The NHIS uses complex survey designs to sample over 35,000 households and 87,500 non-institutionalized individuals annually. The NHIS uses a face-to-face interview format to obtain responses from randomly selected individuals across the 50 states in the U.S. and the District of Columbia [34]. It uses a multistage sampling unit, drawing samples in succession from primary sampling units (counties, metropolitan statistical areas, or contiguous small counties), secondary sampling units (area and permit segments), and addresses, and households [34]. The average yearly household response rate is 70% [35]. Blacks, Asians, and Hispanics are oversampled to increase the precision of the estimates [34]. The sampling response rates vary from 13 to 24% across the years pooled in this study [34].

### 2.2. Inclusion and exclusion variables

**Fig 1:**
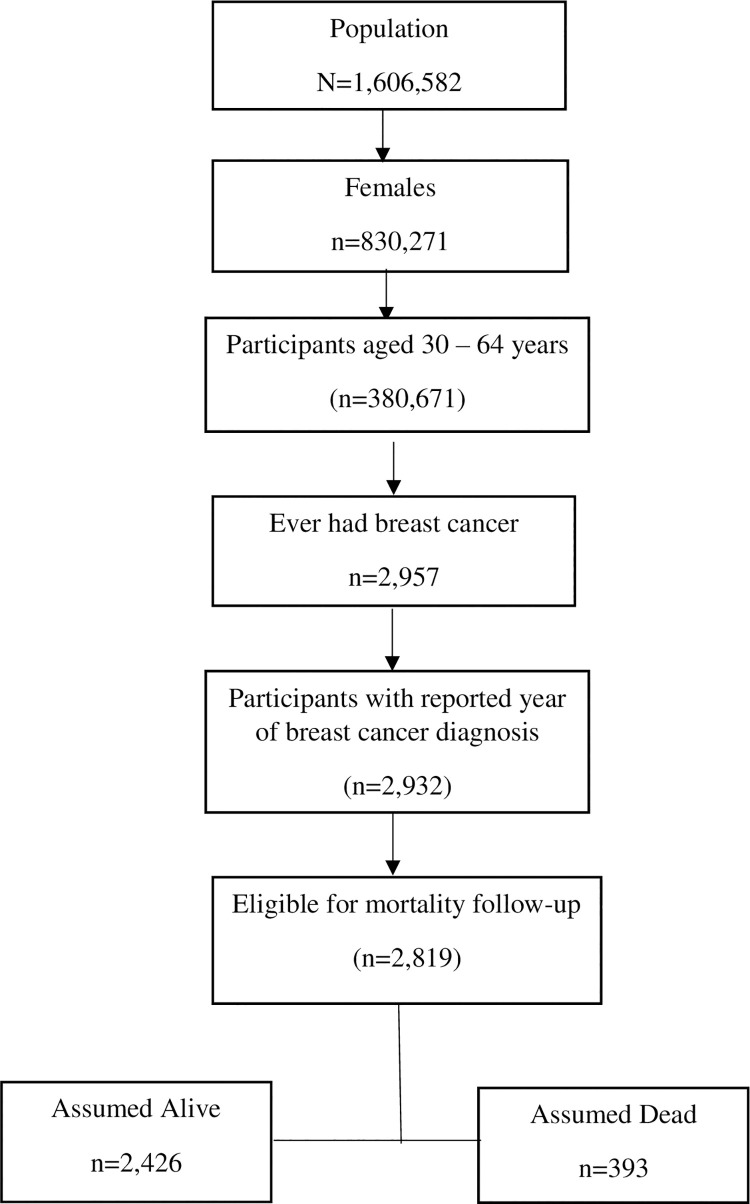
Study population with inclusion and exclusion criteria, NHIS 1998–2014.

The total sample population across the 17 years was 1,606,582 (Fig 1). We restricted the data to females (n = 830,271), and those aged 30–64 years (n = 380,641). We excluded women less than 30 years as breast cancer at ages less than 30 years are fewer [36] and mostly linked with hereditary cancer syndromes–a clinical diagnosis of persons with mutated genes that increases their likelihood for multiple cancers [37]. Women aged 65 years and older were excluded as they may be more likely to be enrolled in Medicare and this may differentially influence health outcomes compared to the younger population [38].

We further restricted the dataset to women with a self-reported diagnosis of breast cancer (n = 2,957), and we excluded those who could not report the year they had the breast cancer diagnosis (n = 25). Finally, we excluded those who were not eligible for mortality follow-up (n excluded = 113). Respondents who had linked mortality status information were defined as being eligible for mortality follow-up. NHIS respondents, aged 18 years and older as at the time of the interview, who provided sufficient identifying information, had their records matched with records from the National Center for Health Statistics (NCHS) [39]. The IPUMS reports the information as mortality status (assumed alive/assumed dead). The final sample size of the study was 2,819, with 393 assumed dead, and 2,426 assumed alive (right censored data).

### 2.3. Outcome variable

The outcome variable was time to death among women with breast cancer diagnoses. We determined time to death as the difference between the year of mortality and the reported year of the breast cancer diagnosis. Censored events represent participants who are assumed alive.

### 2.4. Predictor variables

The main predictors were psychological distress and self-rated health. Psychological distress was computed using the Kessler-6 Scale [40, 41]. The Kessler-6 scale is a validated measure that screens for non-specific anxiety, depression, and mental illness collectively referred to as psychological distress [40, 41]. The NHIS respondents were asked six questions: "Within the last 30 days, how often did you feel (1) worthless, (2) sad, (3) restless, (4) hopeless, (5) everything was an effort, and (6) your feelings interfered with life?" Consistent with the Kessler-6 scoring, each response was measured on a 5-point Likert scale from 1 to 5 representing not at all, a little of the time, some of the time, most of the time, and all the time, respectively. These scores were recoded to 0 to 4 with scores of 13 and higher representing respondents with psychological distress and scores of 0 to 12 representing those without psychological distress. Self-rated health has been reported in earlier studies as a predictor of mortality [26, 42]. For this study, self-rated health was measured as a categorical variable. Participants answered the question, "would you say your health is excellent, very good, good, fair, or poor"? The items chosen by the respondent were reported verbatim on a 5-point Likert scale. For this study, self-rated health was recoded into three groups: poor, fair, and good to excellent [53]. For brevity, “good-to-excellent” is hereafter referred to as “good”.

### 2.5. Potential confounders

For this study, age, race/ethnicity, educational attainment, marital status, health coverage, and poverty income ratio were selected as a priori confounders consistent with the extant literature [27–29]. Also, we selected three measures of health care access as these variables may influence health outcomes. These self-reported variables were the presence of a usual place of care (categorized as yes, no, or missing), perceived affordability of care (categorized as affordable, not affordable, or missing), and delayed appointments (defined as delaying care due to difficulties in getting a timely appointment and ultimately categorized as yes, no, and missing). Additionally, the year of the survey was selected as a potential confounder since breast cancer treatment and survival have improved over the period of study [43].

### 2.6. Data analysis

Missing data were present in all the predictor and confounding variables with the only exception being the age of the study participants (Appendix 1 in S1 File). Across all the variables, the missingness ranged from 0.1% to 13.3%. We performed multiple imputations to provide unbiased estimates for the missing data [44, 45]. First, we determined if the missingness was completely at random using Little’s Covariate-Dependent Missingness test [46]. A p-value of less than 0.05 suggests that the missingness was not at random. For this study, the p-value was 1.0 suggesting the presence of randomness. Next, we performed multiple imputations by chained equation (MICE) using ten iterations and inserted binary logit and ordinary logit functions as appropriate to the variables [47]. Estimates were rounded for all categorical variables. To assess the consistency of the multiply imputed data, we computed the frequency distribution and assessed the correlation between the multiply imputed and raw data using the Cramer V and Kendall tau coefficients for binary and ordinal data, respectively. Across all the assessed variables, the correlation coefficient ranged from 0.95 to 1.00, showing the adequacy of the missing data imputation analysis [45] (Appendix 1 in S1 File).

We reported the frequency distribution in the total sample and among those assumed dead. Also, we assessed the difference across the groups using the log-rank test. Statistical significance was set at a p-value <0.05. We performed a survey-weighted Cox Proportional Hazard Regression, reporting the unadjusted and adjusted hazard ratio (mortality risk ratio) with significance set at a 95% confidence interval. The survey weights were obtained by dividing the sample weight variable by the pooled number of years [48]. We performed a sensitivity analysis by restricting the data to those diagnosed with breast cancer within five years of the NHIS interview (n = 1,395). The 5-year period is the commonly used cancer interval that identifies survivors of specific cancer types [49]. Also, in the setting of breast cancer, the five-year period reflects the cumulative period in which women with breast cancer would have been exposed to surgery, chemotherapy, radiotherapy, and other medical interventions. We were interested in assessing if, within the 5 years, the relationship between mortality and psychological distress and self-rated health will be consistent with the main results. Additionally, we were interested in assessing if the findings were similar among women aged 65 years and older, and we conducted a separate analysis among this cohort (n = 3,618). Data analysis was performed using both STATA version 16 [50] and SAS version 9.4/SAS Studio version 3.71 [51].

### 2.7. Ethical concern

This research used the IPUMS-NHIS, a publicly available de-identified data [52]. Based on the guidance from the Office of Research Protections and Integrity (ORPI) of the University of North Carolina at Charlotte, secondary data analysis of de-identified data that is publicly available does not require IRB approval [53]. Hence, informed consent was not required for this study.

## 3. Results

In this study, most women were non-Hispanic Whites (80%), aged between 50–69 years (47%), married (52%), and had a bachelor’s degree or higher (32%). About 12% of the sample population lived below the poverty line, and about 7% had no healthcare coverage. Also, about 8% of the sample population reported that they had no usual place of care, 11% reported that care was not affordable and 3% reported that they had delayed appointments. Approximately 6% were classified as having psychological distress based on the self-reported scale. Also, 19% and 8% of the sample population rated their health as fair and poor, respectively (Table 1).

**Table 1:** Frequency distribution and association between mortality status and psychological distress, self-rated health, sociodemographic characteristics, and measures of health care access.

Variable	Total Population (N = 2,819)	Dead (n = 393)	Alive (n = 2,426)	Log-Rank Test
	Unweighted Frequency (Weighted Percentage)	Unweighted Frequency (Weighted Percentage)	Unweighted Frequency (Weighted Percentage)	p-value*
**Psychological Distress**				
Present	174 (5.9)	40 (10.2)	134 (5.2)	<0.001
Absent	2,645 (94.1)	353 (89.8)	2,292 (94.8)	
**Self-Rated Health**				
Poor Health	232 (7.5)	74 (18.6)	158 (5.8)	<0.001
Fair Health	561 (18.6)	107 (26.9)	454 (17.2)	
Good Health**	2,026 (73.9)	212 (54.5)	1,814 (77.0)	
**Age Categories**				
30–39 years	141 (4.4)	17 (3.6)	124 (4.6)	<0.001
40–49 years	566 (19.6)	78 (18.1)	488 (19.8)	
50–59 years	1,301 (46.7)	185 (48.7)	1,116 (46.3)	
60–64 years	811 (29.3)	113 (29.6)	698 (29.2)	
**Educational Attainment**				
Less than High School	343 (10.7)	78 (19.1)	265 (9.3)	<0.001
High School or Equivalent	787 (27.2)	115 (28.8)	672 (27.0)	
Some College	844 (30.2)	107 (27.2)	737 (30.7)	
Bachelor’s and higher	845 (31.9)	93 (24.9)	752 (33.0)	
**Marital Status**				
Never Married	287 (9.7)	49 (11.3)	238 (9.4)	<0.001
Divorced/Separated/Widowed	1,103 (38.9)	162 (41.5)	941 (38.5)	
Married	1,429 (51.5)	182 (47.1)	1,247 (52.2)	
**Race/Ethnicity**				
Non-Hispanic Blacks	380 (10.2)	78 (15.3)	302 (9.3)	<0.001
Hispanics	273 (6.3)	26 (4.4)	247 (6.6)	
Other Races	134 (3.5)	16 (3.4)	118 (3.6)	
Non-Hispanic Whites	2,032 (80.0)	273 (76.9)	1,759 (80.5)	
**Poverty-Income Ratio**				
Below PIR	387 (12.2)	76 (19.1)	311 (11.0)	<0.001
At or Above PIR	2,432 (87.8)	317 (80.9)	2,115 (89.0)	
**Health Coverage Status**				
No medical insurance	228 (7.1)	32 (8.3)	196 (6.8)	<0.001
Has medical insurance	2,591 (92.9)	361 (91.7)	2,230 (93.2)	
**Usual place of care**				
No usual place	230 (8.4)	21 (6.0)	209 (8.8)	<0.001
Have a usual place	2,589 (91.6)	372 (94.0)	2,217 (91.2)	
**Affordable care**				
Not affordable	296 (10.5)	42 (11.3)	254 (10.4)	<0.001
Affordable	2,523 (89.5)	351 (88.7)	2,172 (89.6)	
**Delayed appointment**				
Delayed appointment	103 (3.3)	9 (2.6)	94 (3.4)	<0.001
No delayed appointment	2,716 (96.7)	384 (97.4)	2,332 (96.6)	

*Log rank test: p<0.05 means there is a significant difference across the groups

**Good Health represents the sample population who described their health status as good, very good, or excellent.

There were statistically significant differences in the mortality patterns across the self-reported psychological distress (p-value<0.001) and self-rated health (p-value<0.001). Among those that were alive, 5% reported having psychological distress while about 10% of those who died reported having psychological distress. Among those who were alive, 6%, 17%, and 77% reported having poor, fair, and good health, respectively, while among those who died, 19%, 27%, and 54% reported having poor, fair, and good health status, respectively. Additionally, there were statistically significant difference in the mortality patterns across age categories (p-value<0.001), educational attainment (p-value<0.001), marital status (p-value<0.001), race/ethnicity (p-value<0.001), poverty-income ratio (p-value<0.001), health coverage status (p-value<0.001), usual place of care (p-value<0.001), affordable care (p-value<0.001), and delayed appointment (p-value<0.001).

In the unadjusted model, those with less than high school had 88% increased mortality risk compared to those with bachelor’s degrees and higher (Hazard Risk (HR): 1.88; 95% CI: 1.39–2.54) (Table 2). Women with a breast cancer diagnosis who were either divorced, separated, or widowed had a 56% increased mortality risk compared to those who were married (HR: 1.56; 95% CI: 1.14–2.15). Compared to non-Hispanic Whites, non-Hispanic Blacks had a 62% increased mortality risk (HR: 1.62; 95% CI: 1.26–2.08). Also, those who live below the poverty line had a 65% increased mortality risk compared to those who live at or above the poverty line (HR: 1.65; 95% CI: 1.29–2.13). Further, with every unit increase in the year of the survey, the mortality risk reduced by 6% (HR: 0.94; 95% CI: 0.92–0.96).

**Table 2:** Unadjusted hazard risk ratios of the predictor variables on the mortality from breast cancer.

Variable	Unadjusted Hazard Ratio (95% CI)
**Psychological Distress**	
Present	**1.74 (1.25–2.42)**
Absent	Ref
**Self-Rated Health**	
Poor Health	**3.35 (2.57–4.38)**
Fair Health	**1.84 (1.45–2.33)**
Good Health	Ref
**Age Categories**	
30–39 years	1.15 (0.69–1.92)
40–49 years	1.26 (0.94–1.68)
50–59 years	1.13 (0.89–1.43)
60–64 years	Ref
**Educational Attainment**	
Less than High School	**1.88 (1.39–2.54)**
High School or Equivalent	1.27 (0.96–1.67)
Some College	1.15 (0.87–1.52)
Bachelor’s and higher	Ref
**Marital Status**	
Never Married	1.21 (0.98–1.50)
Divorced/Separated/Widowed	**1.56 (1.14–2.15)**
Married	Ref
**Race/Ethnicity**	
Non-Hispanic Blacks	**1.62 (1.26–2.08)**
Hispanics	0.77 (0.51–1.15)
Other Races	1.14 (0.69–1.89)
Non-Hispanic Whites	Ref
**Poverty-Income Ratio**	
Below PIR	**1.65 (1.29–2.13)**
At or Above PIR	Ref
**Health Coverage Status**	
No medical insurance	0.87 (0.60–1.25)
Has medical insurance	Ref
**Usual place of care**	
No usual place	0.70 (0.45–1.09)
Have a usual place	Ref
**Affordable care**	
Not affordable	0.99 (0.72–1.37)
Affordable	Ref
**Delayed appointment**	
Delayed appointment	0.61 (0.31–1.18)
No delayed appointment	Ref
**Year of Survey**	**0.94 (0.92–0.96)**

Also, in the unadjusted hazard model, respondents with self-reported psychological distress had a 74% increased mortality risk as compared to those without self-reported psychological distress (HR: 1.74; 95% CI: 1.25–2.42) (Table 2). After adjusting for the sociodemographic variables and the measures of health care access, the mortality risk was attenuated but remained significant (Adjusted Hazard Ratio (aHR): 1.46; 95% CI: 1.02–2.09) (Table 3). Also, respondents who rated their health as poor and fair had 235% (HR: 3.35; 95% CI: 2.57–4.38), and 84% (HR: 1.84; 95% CI: 1.45–2.33) increased mortality risk respectively, as compared to those who rated their health as good (Table 2). After adjusting for the sociodemographic variables and the measures of health care access, the mortality risks were unchanged (Poor Health aHR: 3.50; 95% CI: 2.61–4.69; Fair Health aHR: 1.83; 95% CI: 1.43–2.35) (Table 3). A sensitivity analysis that restricted the analysis to those who were diagnosed with breast cancer five years or less from the time of the survey showed that psychological distress was associated with elevated though non-significant, mortality risks (aHR: 1.19; 95% CI: 0.70–2.02). Also, the sensitivity analysis showed that poor and fair self-rated health was significantly associated with 2.6 (95% CI: 1.62–4.19) and 1.5 (95% CI: 1.00–2.16) times increased mortality risks, respectively (Table 3). A separate analysis conducted among women aged 65 years and older with breast cancer diagnosis showed a similar pattern of association. Compared to women without self-reported psychological distress, women with self-reported psychological distress had a 47% increased mortality risk (AHR: 1.47; 95% CI: 1.07–2.01). Compared to women aged 65 years and older with good self-rated health, women with poor and fair self-rated health had 111% (AHR: 2.11; 95% CI: 1.75–2.55) and 57% (AHR: 1.57; 95% CI: 1.36–1.80) increased mortality risk, respectively (Appendix 2 in S1 File).

**Table 3:** Adjusted hazard ratio of the effect of psychological distress and self-rated health on time to death among women aged 30–64 years diagnosed with breast cancer (N = 2,817) and a sensitivity analysis on those diagnosed five years or less from the time of the survey (N = 1,395).

Variable	Adjusted Hazard Ratio (95% CI)	Adjusted Hazard Ratio (95% CI) (Sensitivity Analysis)
**Model 1: Psychological Distress**		
Present	**1.46 (1.02–2.09)**	1.19 (0.70–2.02)
Absent	Ref	Ref
**Model 2: Self-Rated Health Status**		
Poor Health	**3.50 (2.61–4.69)**	**2.60 (1.62–4.19)**
Fair Health	**1.83 (1.43–2.35)**	**1.47 (1.00–2.16)**
Good Health	Ref	Ref

Model 1: Modeling the adjusted hazard of mortality from psychological distress; Model 2: Modeling the adjusted hazard of mortality from self-rated health; All the models are adjusted for age, race/ethnicity, educational attainment, marital status, health coverage, available care, affordable care, accessible care, poverty income ratio, and year of the survey. Sensitivity analysis was performed by restricting the data to those who were diagnosed with breast cancer five years or less from the time of the survey (n = 1,395).

**Fig 2:**
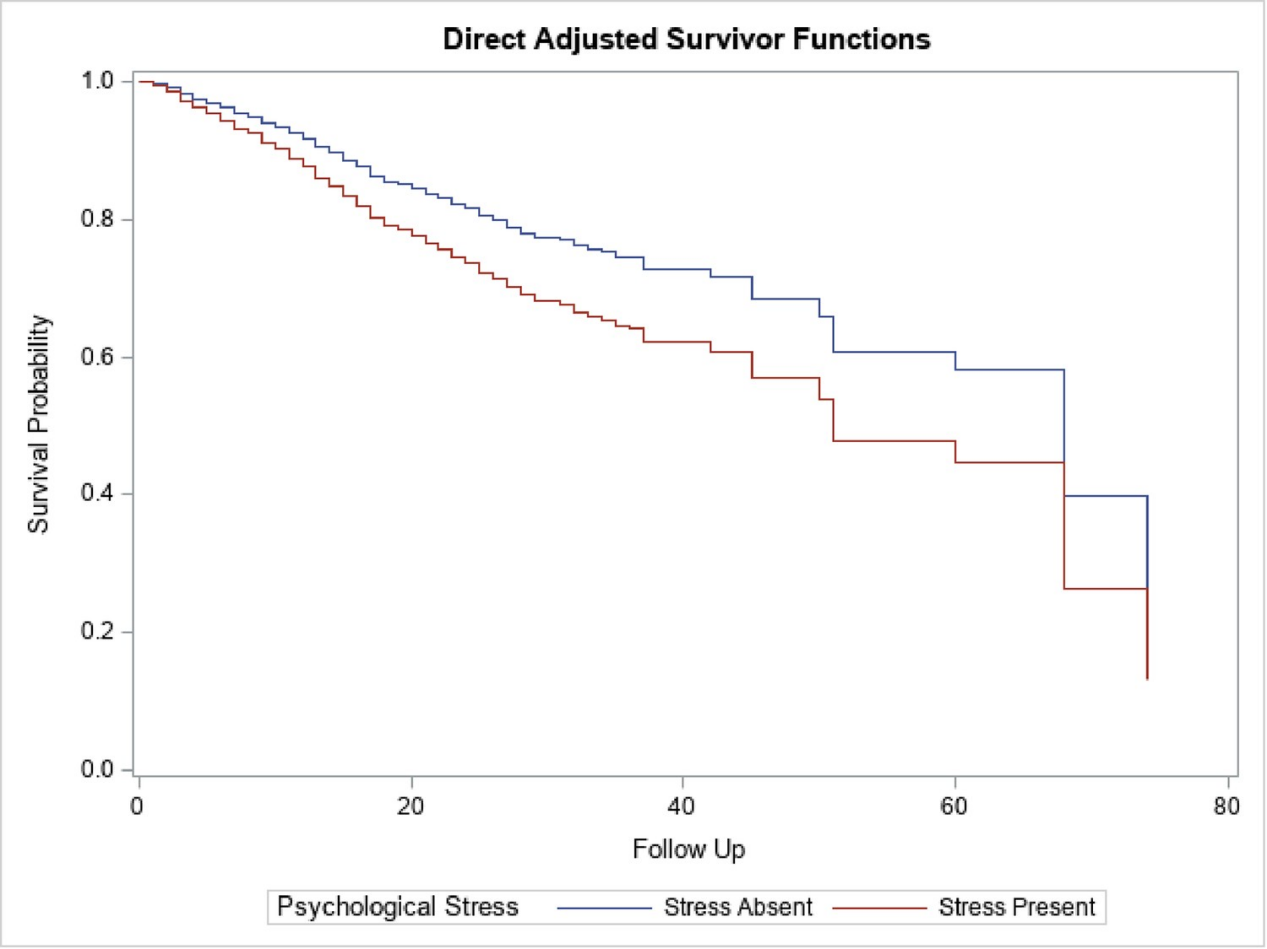
Cox-Proportional Hazard model stratified by symptoms of psychological stress. Median (Q1, Q3) survival time among the population with psychological stress was 32 years (17 years, 50 years) while median survival time among those without psychological stress was 60 years (32 years, 68 years) The regression model was adjusted for age, race/ethnicity, educational attainment, marital status, poverty income ratio, health coverage, delayed appointment, usual place of care, affordable care, and year of survey.

**Fig 3:**
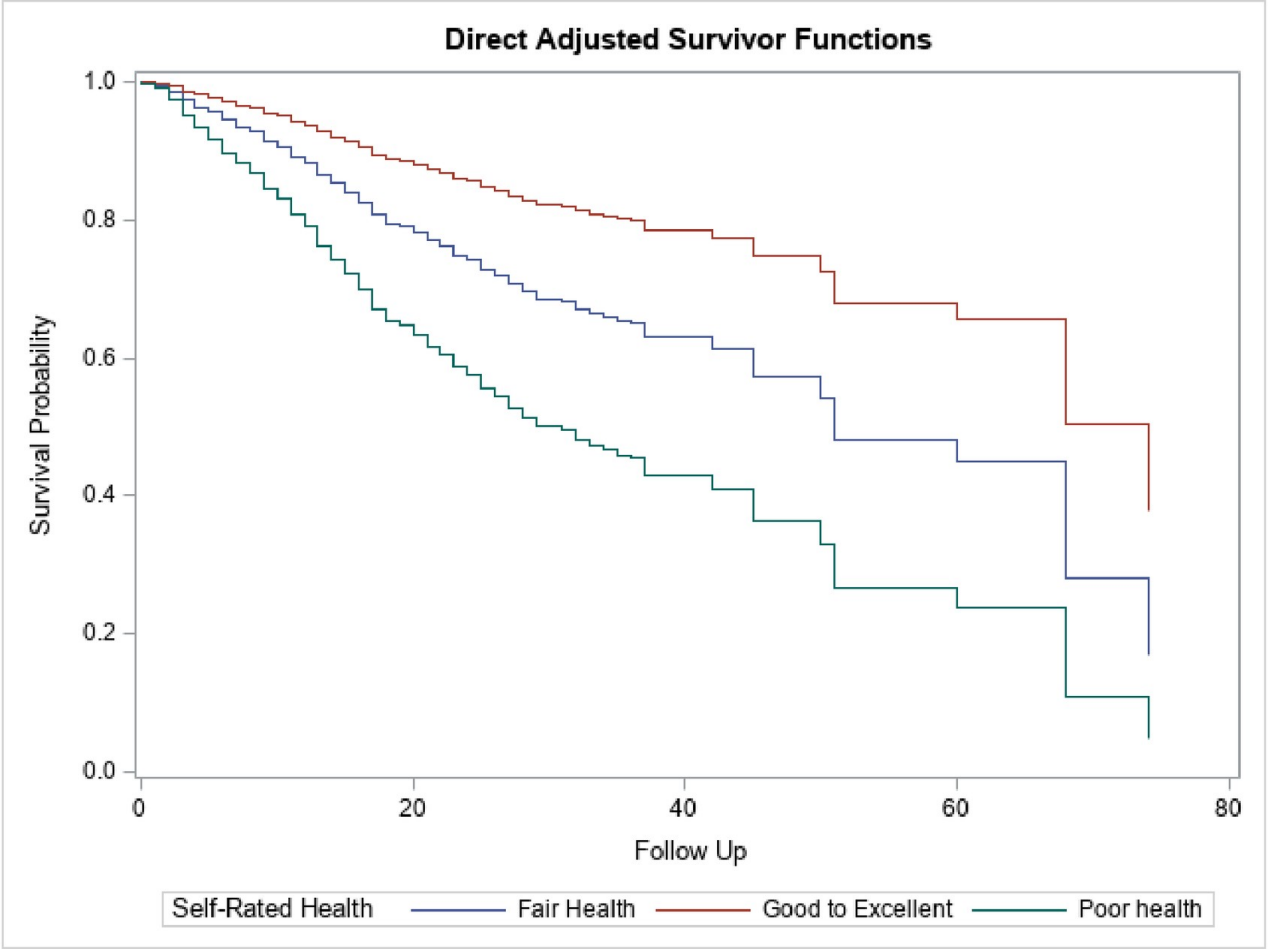
Cox-Proportional Hazard model stratified by symptoms of psychological stress. The median survival times among the population with poor self-rated health was 28 years (15 years, 60 years), fair self-rated health was 45 years (20 years, 74 years), and good self-rated health was 68 years (37 years, 68 years). The regression model was adjusted for age, race/ethnicity, educational attainment, marital status, poverty income ratio, health coverage, delayed appointment, usual place of care, affordable care, and year of survey.

Women with self-reported psychological distress had a significantly lower survival rate compared to women without psychological distress (Fig 2). The adjusted median survival time among women with and without self-reported psychological distress was 32 years and 60 years, respectively. Furthermore, the survival times were lowest among women with self-rated poor health and highest among women with self-rated good health (Fig 3). The median survival time was 28, 45, and 68 years among women who rated their health as poor, fair, and good respectively.

## 4. Discussion

In this study, about 1 in 17 women with breast cancer diagnoses were evaluated as having psychological distress. While this low proportion of psychological distress may reflect the immense resilience women with breast cancer demonstrate, more than one-quarter of these women rated their health as being fair or poor. Women with breast cancer diagnoses who were classified as having psychological distress from their self-reported responses had statistically significant increased risks of mortality and lower survival times compared to those who were classified as not having psychological distress. Also, women who rated their health as fair or poor had significantly increased risks of mortality and lower survival times compared to those who rated their health as good.

Few studies have assessed the relationship between psychological distress and mortality from breast cancer. Hamer et al. [54], using the General Health Questionnaire to define psychological distress, reported that psychological distress was associated with a 27% increased risk of all cancer deaths among the Scottish population. In contrast to the study conducted by Hamer et al. [54] whose population included males and females with any cancer history, our study focused on women with breast cancer diagnoses. Furthermore, we report a 46% increased mortality risk from psychological distress, defined using the Kessler-6 scale [40, 41], among U.S. women with breast cancer diagnoses. Gilbar [55], using the Brief Symptoms Inventory to assess psychological distress among hospitalized patients in Israel, reported that women who died from breast cancer had higher psychological distress scores than those who survived. While Gilbar [55] did not compute the mortality risk estimate associated with psychological distress, Gilbar’s 27-year old study provided early indications on the association between psychological distress and breast cancer-related mortality. More recently, Lu et al. [56] reported that psychological distress diagnosed within one year before and after breast cancer diagnosis was associated with a 30% increased mortality risk from cervical cancer among Swedish women.

This study highlights the need for screening and treatment for psychological distress among women with breast cancer. Several studies have documented the pathway connecting psychological distress and mortality [11, 16–21] but the paucity of literature on the impact of early and consistent screening for psychological distress and its relationship with breast cancer survivorship is alarming. Most screening tools like the Kessler-6 scale [40, 41], Perceived Stress Scale [57], the Brief Symptoms Inventory-18 [58] amongst others [59], rely on self-reported measures, and they represent low-cost measures that health care providers can incorporate in breast cancer treatment regimen. Additionally, it is unknown to what extent psychotherapy interventions reduce psychological distress among breast cancer patients and to what extent it improves survivorship. This study reports that about 6% of the women with breast cancer had a self-reported assessment of psychological distress. However, the possibility exists that the proportion would be higher if the assessment were a hospital-based survey as opposed to the population-based survey of the NHIS.

In this study, self-reported fair and poor health status was also a statistically significant indicator of mortality among women with breast cancer. The relationship between self-rated health and mortality has been extensively studied [26, 60, 61], although those that focus on breast cancer-related survival are few [62]. Irrespective of the disease, self-rated health has been reported as an independent predictor of mortality [61]. A conceptual explanation of self-rated health is that atypical bodily sensations during illness are interpreted by an individual’s knowledge of what good health should represent. A judgment of an individual’s health quality is made by comparing the current perceived quality of health and what it should be [61]. Such a rating will be more accurate if the individual is aware of coexisting health conditions and the prognosis of such disease [61].

This study has its limitations. The NHIS is a cross-sectional study and causation cannot be established. Also, the temporal order of psychological distress or self-rated health and breast cancer diagnoses cannot be determined. Hence, we cannot imply causation or infer beyond the observed relationship documented in this study. Further, nondifferential misclassification from self-reports of the exposure variables is likely [63]. We defined psychological distress using a validated instrument [40, 41] which captures non-specific anxiety, depression, and severe mental illness. Our definition of psychological distress differs from other forms of stress from life events, chronic stress, acute stress, daily stress, or traumatic stress although these stress types may be conceptually related [64]. Also, nondifferential misclassification of the outcome is less likely as mortality status was obtained from the NDI, which registers all deaths in the U.S. Although non-response bias cannot be eliminated, the NHIS response rate is comparable to the response rates of other national surveys such as the National Health and Nutritional Examination Surveys (NHANES) [65] and Behavioral Risk Factors Surveillance System (BRFSS) [66]. Additionally, control for confounding was limited to the questions asked by the NHIS. A family history of breast cancer, cancer stage at diagnosis, type of cancer treatment, nulliparity, duration and severity of co-morbid illnesses, performance status, and the presence of cancer recurrence are potential confounders that were not assessed in this population-based study. These clinically relevant variables may affect the result of this study. Despite these limitations, this study has several strengths. This population-based study provides an estimate of risk distributions in the reference population. This study represents one of the few studies that reported the association of self-reported symptoms of psychological distress and self-rated health on survival times among women with breast cancer. To our knowledge, no publicly available study in the has reported the mortality risk associated with psychological distress and fair or poor self-rated health among women with breast cancer diagnoses. Furthermore, this study is a nationally representative sample of women with breast cancer and the results can likely be generalized to breast cancer patients across the U.S.

## 5. Conclusion

Self-reported psychological distress and fair and poor self-rated health are associated with reduced survival times among women with breast cancer diagnoses. Screening and treatment for psychological distress among women with breast cancer diagnoses may improve the quality of life and cancer survivorship. This study presents areas of future research as breast cancer stage, aggressiveness, recurrence, and treatment type might play a role in the occurrence of psychological distress, poor self-rated health, and mortality. As the frontiers of breast cancer screening, diagnosis, and treatment keep expanding, addressing psychological distress among women with breast cancer diagnosis may further improve their quality of life.

## Supporting information

S1 Data Data was downloaded from IPUMS NHIS. Compressed data for this study available at https://doi.org/10.6084/m9.figshare.16723069.v1.
(DTA)

S1 File (DOCX)
