# Supplementary material for: Evaluating the association of self-reported psychological distress and self-rated health on survival times among women with breast cancer in the U.S
Source: PLoS One. 2021 Dec 1;16(12):e0260481. doi: 10.1371/journal.pone.0260481 (PMC8635381; doi:10.1371/journal.pone.0260481)
Supplement: S1 File — (DOCX) [file pone.0260481.s002.docx]

Appendix 1: Correlation between the frequency distribution of the raw and multiply imputed data

| Variable | Raw Data  (N=2,819) | Multiply Imputed Data* (N=2,819) | Correlation Coefficient |
| --- | --- | --- | --- |
|  | Frequency (%) | Frequency (%) | (p-value) |
| **Psychological Distress***** |  |  |  |
| Present | 171 (6.1) | 174 (6.2) | 0.992 (<0.001) |
| Absent | 2,613 (92.7) | 2,645 (93.8) |  |
| Missing Data | 35 (1.2) | NA |  |
| **Self-Rated Health **** |  |  |  |
| Poor Health | 231 (8.1) | 232 (8.2) | 0.998 (<0.001) |
| Fair Health | 561 (19.9) | 561 (19.9) |  |
| Good Health | 2,025 (71.8) | 2,026 (71.9) |  |
| Missing Data | 2 (0.1) | NA |  |
| **Educational Attainment**** |  |  |  |
| Less than High School | 342 (12.1) | 343 (12.2) | 0.997 (<0.001) |
| High School or Equivalent | 786 (27.9) | 787 (27.9) |  |
| Some College | 841 (29.8) | 844 (29.9) |  |
| Bachelor’s and higher | 845 (30.0) | 845 (30.0) |  |
| Missing Data | 5 (0.2) | NA |  |
| **Marital Status**** |  |  |  |
| Never Married | 287 (10.2) | 287 (10.2) | 0.995 (<0.001) |
| Divorced/Separated/Widowed | 1,098 (38.9) | 1,103 (39.1) |  |
| Married | 1,427 (50.6) | 1,429 (50.7) |  |
| Missing Data | 7 (0.3) | NA |  |
| **Race/Ethnicity**** |  |  |  |
| Non-Hispanic Blacks | 376 (13.3) | 380 (13.5) | 0.998 (<0.001) |
| Hispanics | 272 (9.7) | 273 (9.7) |  |
| Other Races | 134 (4.8) | 134 (4.8) |  |
| Non-Hispanic Whites | 2,032 (72.0) | 2,032 (72.1) |  |
| Missing Data | 5 (0.2) | NA |  |
| **Poverty-Income Ratio***** |  |  |  |
| Below PIR | 350 (12.4) | 387 (13.7) | 0.949 (<0.001) |
| At or Above PIR | 2,093 (74.3) | 2,432 (86.3) |  |
| Missing Data | 376 (13.3) | NA |  |
| **Health Coverage Status***** |  |  |  |
| No medical insurance | 228 (8.1) | 228 (8.1) | 1.00 (<0.001) |
| Has medical insurance | 2,586 (91.7) | 2,591 (91.9) |  |
| Missing Data | 5 (0.2) | NA |  |
| **Usual place of care***** |  |  |  |
| No usual place | 230 (8.2) | 230 (8.2) | 1.00 (<0.001) |
| Have a usual place | 2,566 (91.0) | 2,589 (91.8) |  |
| Missing Data | 23 (0.8) | NA |  |
| **Affordable care***** |  |  |  |
| Not affordable | 296 (10.5) | 296 (10.5) | 1.00 (<0.001) |
| Affordable | 2,520 (89.4) | 2,523 (89.5) |  |
| Missing Data | 3 (0.1) | NA |  |
| **Delayed appointment***** |  |  |  |
| Delayed appointment | 103 (3.7) | 103 (3.7) | 1.00** (<0.001) |
| No delayed appointment | 2,697 (95.7) | 2,716 (96.3) |  |
| Missing Data | 19 (0.7) | NA |  |
| Little’s CDM test**** |  |  | p-value: 1.000 |

*Covariates used for the multiple imputation model and the Little’s CDM test include the six variables that defined psychological distress, self-rated health, educational attainment, marital status, race/ethnicity, poverty-income ratio, health coverage, usual place for care, affordable care, and delayed appointment

**Kendall tau coefficient computed since categorical variables are ordered

***Cramer V coefficient computed for categorical variables that are not ordered.

**** CDM: Covariate-Dependent Missingness; p-value <0.05 suggests not missing at random and p-value >= 0.05 suggests that the assumption of completely missing at random may hold.

Appendix 2: Adjusted hazard ratio of the effect of psychological distress and self-rated health on time to death among women aged 65 years and older diagnosed with breast cancer (N=3,168)

| Variable | Adjusted Hazard Ratio  (95% CI) |
| --- | --- |
| **Model 1: Psychological Distress**  Present  Absent | **1.47 (1.07 – 2.01)**  Ref |
| **Model 2: Self-Rated Health Status**  Poor Health  Fair Health  Good Health | **2.11 (1.75 – 2.55)**  **1.57 (1.36 – 1.80)**  Ref |

Model 1: Modeling the adjusted hazard of mortality from psychological distress; Model 2: Modeling the adjusted hazard of mortality from self-rated health; All the models are adjusted for age, race/ethnicity, educational attainment, marital status, health coverage, available care, affordable care, accessible care, poverty income ratio, and year of the survey.
